# A review of the literature on the use of CRISPR/Cas9 gene therapy to treat hepatocellular carcinoma

**DOI:** 10.32604/or.2023.044473

**Published:** 2024-02-06

**Authors:** ELHAM AMJAD, RAFFAELE PEZZANI, BABAK SOKOUTI

**Affiliations:** 1 Biotechnology Research Center, Tabriz University of Medical Sciences, Tabriz, 5165665813, Iran; 2 Phytotherapy Lab, Endocrinology Unit, Dipartimento di Medicina (DIMED), University of Padova, Via Ospedale 105, Padova, 35128, Italy; 3 Associazione Italiana Per La Ricerca Oncologica Di Base, Associazione Italiana Per La Ricerca Oncologica Di Base, Padova, 35128, Italy

**Keywords:** CRISPR/Cas9 system, Gene therapy, Tumor, Hepatocellular carcinoma, Liver cancer, Gene editing

## Abstract

Noncoding RNAs instruct the Cas9 nuclease to site-specifically cleave DNA in the CRISPR/Cas9 system. Despite the high incidence of hepatocellular carcinoma (HCC), the patient’s outcome is poor. As a result of the emergence of therapeutic resistance in HCC patients, clinicians have faced difficulties in treating such tumor. In addition, CRISPR/Cas9 screens were used to identify genes that improve the clinical response of HCC patients. It is the objective of this article to summarize the current understanding of the use of the CRISPR/Cas9 system for the treatment of cancer, with a particular emphasis on HCC as part of the current state of knowledge. Thus, in order to locate recent developments in oncology research, we examined both the Scopus database and the PubMed database. The ability to selectively interfere with gene expression in combinatorial CRISPR/Cas9 screening can lead to the discovery of new effective HCC treatment regimens by combining clinically approved drugs. Drug resistance can be overcome with the help of the CRISPR/Cas9 system. HCC signature genes and resistance to treatment have been uncovered by genome-scale CRISPR activation screening, although this method is not without limitations. It has been extensively examined whether CRISPR can be used as a tool for disease research and gene therapy. CRISPR and its applications to tumor research, particularly in HCC, are examined in this study through a review of the literature.

## Introduction

Cancer’s aberrant cells infect neighboring organs and can then spread to other body parts, making it a worldwide health hazard. It is the second top killer throughout the globe. One in six people is diagnosed with cancer at some point in their lives, and the disease claims the lives of more than 700,000 people annually out of a total annual global cancer toll of 20 million [1]. The most common kind of liver cancer is hepatocellular carcinoma (HCC). Several risk factors, such as hepatitis B and C, cirrhosis infections, alcoholism, and non-alcoholic fatty liver disease or NAFLD, can develop HCC. To treat HCC successfully, surgeons recommend resection of the cancer. HCC has an aggressive growth pattern and late symptoms, so most patients are left untreated because their disease is late diagnosed and at an advanced stage when they are diagnosed. A median survival rate of 9 months is available only for patients with advanced HCC, while a 10% overall survival rate is available for patients with advanced HCC [2].

There are many different treatment strategies for HCC, each with its limitations, which gene therapy tries to address [3,4]. Some of these limitations include i) surgery and transplantation with limited donor shortages, ii) strict selection criteria, iii) the risks of major surgery as well as post-operational recurrence, iv) local ablative therapies such as radiofrequency ablation effective only for small tumors, v) chemotherapy and radiation therapy with numerous side effects, vi) targeted therapies and immunotherapy with potential common resistance, and vii) advanced liver diseases such as cirrhosis or advanced liver disease with limited treatment options [5–7].

By offering increased specificity, lower systemic toxicity, potential applicability regardless of liver function status, and potentially synergistic effects with existing treatments, gene therapy seeks to overcome these limitations [8–11]. Despite this, gene therapy is still a very experimental approach to HCC and comes with its own set of challenges, including delivery mechanisms, off-target effects, and integration with current treatment paradigms [12–15].

HCC is among the diseases that CRISPR/Cas9 may revolutionize therapeutically through its ability to edit genes. HCC therapy has some limitations, such as delivery and specificity, off-target effects, the complicated genetic landscape of HCC, immune responses, limited repair mechanisms, heterogeneity of the tumor, and regulatory and ethical issues. Addressing these challenges is crucial to fully exploiting CRISPR/Cas9 as a therapeutic tool for HCC. In addition to developing strategies for mitigating these limitations, researchers are actively refining the technology.

From the defense mechanisms used by bacteria to combat viral infections, CRISPR/Cas9 originates. A region known as CRISPR (Clustered Regularly Interspaced Short Palindromic Repeats) is used by bacteria to store viral DNA segments. During the invasion, the bacteria transcribe viral DNA segments, cleaved by a DNA-cleaving enzyme called Cas9. When these segments are cleaved, the bacteria neutralize them. Single-guide RNA (sgRNA) binds a specific message to target genes using DNA sequences. Because of this guide, Cas9 will eventually reach the correct location on the genome. It is possible to design a sgRNA that matches the sequence of a specific gene to target that gene. These types of sgRNA are typically 20 nucleotides long. Once inside the cell, Cas9 binds to the short guide RNA. This complex scans the cell in response to detecting a matching DNA sequence. Protospacer Adjacent Motif (PAM) is a short adjacent motif used by Cas9-sgRNA to identify targets. The PAM sequence is the only mechanism by which Cas9 binds and cleaves DNA. Once the Cas9 protein recognizes the target DNA sequence, the DNA is cut exactly where it needs to be. As the cell tries to repair itself, it cleaves the DNA strand as soon as it sees it. CRISPR/Cas9 can be used to knock out genes, insert genes, repress or activate genes, and repair this damage using nonhomologous end joining (NHEJ) and homology-directed repair (HDR) [16].

A gene therapy treatment replaces or corrects undesirable or dysfunctional genes in a cell by replacing or correcting them. The term is generally associated with human gene therapy but can also apply to plant and animal gene therapy [17].

Genetically, HCC is a disease that can be directly targeted and modified using gene-editing technologies like CRISPR/Cas9. As a result, patients may be able to receive more personalized and effective therapies than they would have been previously.

It is important to note that HCC is influenced by genetic and non-genetic factors, including chronic hepatitis B and C infection, alcohol consumption, and exposure to aflatoxins [18–20]. There are some key genes and pathways that are commonly mutated in HCC, including TP53, CTNNB1, and the Ras/Raf/MAPK pathway, among others [21,22].

There are several ways gene editing could be used to treat HCC, including 1) correcting mutations that cause HCC cells to multiply and survive, 2) gene silencing, which increases the malignancy of HCC when overexpressed, 3) activating tumor suppressors, which normally prevent the formation of tumors, and 4) introducing specific mutations into healthy liver cells to model and discover drugs.

Moreover, CRISPR can modify gene expression without altering DNA sequences and editing DNA sequences. It is also possible to activate or repress transcription by fusing “dead” Cas9s to transcriptional activators or repressors [23–25].

Since genetic mutations and undesired expressions of specific genes cause many hereditary disorders, gene therapy shows enormous potential for treating and even curing several of these conditions. Consequently, gene therapy has received more attention from the scientific and pharmaceutical communities [26].

In prokaryotic organisms, it was discovered that CRISPR was present in bacteria and archaea. Although nucleotide sequences of this type can be found in many bacteria and archaea, scientists have failed to recognize its purpose for almost a decade. These unique DNA sequences were once used for genotyping bacteria as a biomarker and a unique feature of a bacterium. As the CRISPR/Cas system, combined with the application of recombination DNA technology, has become increasingly recognized by scientists over the past few years, prokaryotic cells have developed adaptive immunity to viruses. The CRISPR/Cas system has gained widespread attention because of its ability to target particular genes for editing within a genome [27].

CRISPR/Cas9 gene therapy has been investigated in several studies for HCC [28]. CRISPR/Cas9, among other things, shows promise as a potential treatment and biomarker for HCC. However, while these results are promising, it’s essential to note that further testing and safety evaluations are required before lab results can be translated to clinically approved treatments [28]. It has been discovered that CRISPR/Cas9-mediated disruption of TERT promoter mutations inhibits tumor growth in xenografts derived from patients [29]. This is one of the most important studies that illustrates the potential of CRISPR/Cas9. Using CRISPR/Cas9, HCC mouse models were able to suppress tumor growth by targeting CTNNB1 [30]. HCC cell proliferation and tumor growth were significantly inhibited through CRISPR/Cas9-mediated silencing of VEGFA. Mice developed liver tumors similar to human HCC using CRISPR/Cas9, suggesting this approach is useful for modeling human liver cancer [31].

We present a narrative review that provides evidence that CRISPR/Cas9 system gene therapy can treat HCC in humans, cell lines, or animal models.

## Materials and Methods

The literature search was limited to original research articles published in English in Scopus and PubMed. Research terms included “CRISPR”, “hepatocellular carcinoma”, “HCC”, “liver cancer” and combinations of these terms.

### Cancer and gene therapy techniques

One of the most exciting aspects of gene therapy is the ability to target cancer’s genetic causes. By identifying and correcting the specific genetic mutations that drive cancer, gene therapy could potentially halt the progression of cancer. The targeted approach may be less toxic and more effective than traditional cancer treatments such as chemotherapy [32].

Gene therapy involves altering cells (*in vitro* or *in vivo*) with genetic material to facilitate a cure. *In vitro* and preclinical animal models, gene therapy agents have been demonstrated to be highly effective [33,34]. The CRISPR/Cas system, zinc finger endonuclease (ZFN), and transcription activator-like effector nuclease (TALEN) are only a few of the tools utilized for gene editing [35].

The following sections will compare CRISPR/Cas9 with ZFN, TALEN, and CRISPR/Cas9. In CRISPR/Cas9, an RNA molecule (sgRNA) guides the Cas9 protein to a specific DNA sequence, causing it to cleave. A number of its unique features include simplicity, versatility, and the possibility of targeting multiple genes simultaneously. Off-target effects can occur as well [36]. Target recognition is based on the presence of the PAM sequence [37]. Various cancers have been researched with ZFN [38]. It can be created by knockouts or introduced with specific mutations. It recognizes and binds to specific DNA sequences by using a protein domain. Once a break is presented in the DNA, the nuclease domain acts as a break agent. The zinc finger domain can be engineered to target desired DNA sequences, but ZFNs have unique features such as high specificity. In addition to modifying T cells to resist cancer therapeutic effects, it can be used in clinical trials to modify T cells. When a specific mutation is known to cause cancer, this treatment is appropriate [38].

In TALEN, DNA sequence recognition is done by a protein domain (from plant pathogens), similar to ZFN, but with high specificity. The nuclease domain then cuts DNA. A TALE domain can be designed to target specific DNA sequences and is more straightforward than ZFN. TALE domains might be more difficult to deliver to cells because they are larger than ZFN and CRISPR. Since it is larger than ZFN and CRISPR, it may prove more challenging to deliver into cells. It can target specific oncogenes or tumor suppressor genes in cancer research [39,40]. Like ZFN, it may target cancers already known to carry targeted mutations [41–43].

The three methods are very precise in combination. While ZFN and TALEN have been considered more specific than early versions of CRISPR/Cas9, advances in CRISPR/Cas9 design have made it more specific. Designing new targets with CRISPR/Cas9 is easier and faster than using ZFNs or TALENS. It can target multiple genes simultaneously (multiplexing) more easily than ZFNs or TALENS. Certain delivery methods, especially viral vectors, are limited by the large size of TALENs. Whether a particular technique is appropriate for a specific type of cancer or mutation depends more on the therapeutic strategy (e.g., knockouts, gene corrections, gene additions) than on the type of cancer. These treatments have, however, been researched for their ability to alter immune cells (like T cells) so that cancer cells can be targeted and destroyed more effectively [38].

Studies using gene-editing techniques, whether *in vitro* or *in vivo*, have demonstrated significant successes in cancer treatment. Some specific examples are listed below. The EML4-ALK fusion gene was targeted by researchers using CRISPR/Cas9 in a study to treat non-small-cell lung cancer (NSCLC). They observed decreased tumor cell viability and increased apoptosis (cell death) when this gene was knocked out in cancer cell lines. As mentioned, the researchers observed a significant reduction in tumor growth in tumor-bearing mice when using CRISPR/Cas9 to target EML4-ALK. ZFNs were used in a study to correct a mutation in the IL2Rγ gene in human stem cells. Mutations of this gene cause severe combined immunodeficiency. After gene correction, the cells showed normal IL2Rγ expression. The ZFNs showed therapeutic potential when transplanted into mice, where they demonstrated normal development of immune cells. T cells with the CCR5 gene disrupted had resistance to HIV infection in another study, which used TALENs to disrupt the CCR5 gene. The PLK1 gene, known to be overexpressed in many tumors, was targeted and disrupted with TALENs in a study focused on cancer. By knocking out this gene in gastric cancer cell lines, cell proliferation and apoptosis were reduced. TALENs were also demonstrated to significantly reduce the growth of tumors when PLK1 was disrupted using TALENs in mice-bearing tumors [44,45].

When comparing CRISPR to other gene editing tools such as ZFNs and TALENs, several factors must be taken into consideration, including its ease of use and design (customization and universality), its speed (e.g., rapid adaptation and parallel editing), its cost effectiveness (simple implementation reduces expense), and its scalability (high-throughput screening and multiplexing), as well as its versatility [46]. Since CRISPR/Cas9-based gene therapy has rapidly developed, it is becoming more flexible in treating human diseases. While CRISPR/Cas9 has these advantages, it is also worth noting that it has limitations, such as the possibility of off-target effects. However, further refinements are constantly being made to increase its effectiveness. CRISPR/Cas9-based gene therapy, including the delivery system, has rapidly modified, so extensive preclinical and clinical trials have been conducted [26].

With CRISPR technology, gene therapy techniques have been revolutionized, particularly in treating cancer and other diseases [44]. In addition to viral vectors and lipid nanoparticles as delivery mechanisms, CRISPR can precisely alter specific DNA sequences, directly addressing the genetic causes of diseases [47,48]. Due to their efficiency in delivering genetic material into cells, adeno-associated viruses (AAVs) are often used for CRISPR delivery [48]. Nevertheless, ensuring the body’s immune system does not neutralize the drugs, affecting their effectiveness, is critical. These vectors are considered safer than viral ones because they encapsulate the CRISPR components, eliminating the risk of immune reactions to viral vectors. Trials can be conducted both *in vivo* and *out vivo*. For example, CRISPR targets and destroys DNA in cancer cells *in vivo*. It is possible to target blood disorders and cancer with CRISPR/Cas9. It is possible to edit cells removed from patients in the laboratory and then reintroduce the modified cells back into the patient [49]. Blood disorders and cancer are other diseases that can be treated. A milestone trial with sickle cell disease, characterized by abnormal hemoglobin, leads to crescent-shaped red blood cells. A gene that restores fetal hemoglobin production has been edited using CRISPR, compensating for the defect in adult hemoglobin common in sickle cell anemia. By editing three genes in patients’ T cells with CRISPR/Cas9, researchers demonstrated CRISPR/Cas9’s potential as a “living drug” in cancer treatment, demonstrating its effectiveness in attacking and killing cancer cells [50]. Despite their importance, these trials and therapies pose challenges related to ethical considerations, regulatory approvals, and ensuring their safety and efficacy in the long run.

Gene therapy is a tool that can be used to treat cancer since most cancers are caused by mutations in genetic information, particularly mutations of oncogenes and epigenetic changes. In both animal models and cancer cell lines, the CRISPR/Cas9 genome editing method has been shown to be efficient in modifying gene sequences [51].

Mutations in genes are well known to contribute to cancer development since these mutations often lead to uncontrollable cell growth, resulting in tumor growth. With CRISPR/Cas9, a sophisticated gene-editing tool, this mutation could potentially be corrected at the DNA level, halting or reversing tumor growth [52]. The treatment focuses on targeting oncogenes, repairing tumor suppressor genes, enhancing immunotherapy, and disrupting resistance mechanisms in cancer therapy. By modifying certain genes, oncogenes are turned into cancer-causing genes, which facilitate cell proliferation and survival by driving the growth of the disease. These oncogenes can be directly targeted and disrupted within cancer cells using CRISPR/Cas9. With CRISPR/Cas9, cancer cells cannot proliferate uncontrollably, so they may stop growing or even shrink as they die by disabling oncogenes. Mutations can inactivate tumor suppressor genes, which control cell growth if you consider repairing tumor suppressor genes. Using CRISPR/Cas9, these mutations can be corrected by substituting a healthy sequence for the mutated sequence, thus restoring their normal functionality. The reactivation of tumor suppressor genes can reduce or contain tumor masses by restoring normal cellular mechanisms that prevent excessive cell division and survival. Immune cells (like T cells) can be modified using CRISPR/Cas9 to improve immunotherapy [53]. For example, a patient’s T cells are harvested, and genes within those cells are modified using CRISPR/Cas9 to target cancer cells more effectively. It has been shown that genetically modified immune cells can identify, attack, and destroy cancer cells more efficiently when reintroduced into the body, overcoming the immune evasion characteristic of cancerous tumors. It has been shown that CRISPR/Cas9 can alter genes involved in drug-resistance pathways inside cancer cells by disrupting resistance mechanisms. This may lead to resistance to therapies, including chemotherapy. By disrupting these resistance mechanisms, CRISPR/Cas9 can decrease tumor growth and prevent relapse by making cancer cells more susceptible to conventional treatments [54].

### CRISPR/Cas9 gene therapy

This RNA-guided DNA targeting tool has been repurposed to edit genomes, disrupt transcriptional activity, modify epigenetics, and image genomics using clustered regularly interspaced short palindromic repeats (CRISPR) and the CRISPR-associated protein 9 (Cas9) [55]. The CRISPR/Cas9 gene, originally discovered as a bacterial immune defense system, has been used to manipulate genes in various types of cells and organisms. In addition to its most famous application for correcting genetic defects, it has been utilized in various other research fields. Several key examples of CRISPR/Cas9 repurposing include Creating Model Organisms (e.g., a mouse model for Duchenne muscular dystrophy (DMD)), Functional Genomics (e.g., identifying genes involved in resistance to cancer therapy), Agriculture (e.g., crop improvement), Gene Drives for Controlling Vector-Borne Diseases (e.g., creating mosquitoes with a gene drive that conferred resistance to malaria parasites), Treating Genetic Disorders (e.g., a clinical trial for Leber congenital amaurosis), HIV Research (as a means to eliminate HIV from infected cells) [56,57]. Researchers can use the technology to precisely modify nearly any genomic sequence by encoding a guide RNA sequence in a fusion protein, which they can then use to inhibit growth or activate oncogenes and cancer silencer genes, as well as research genes involved associated with disease development and progression [58]. Programmable endonuclease technology allows researchers to target numerous genomic loci at once, allowing them to study the function of multiple genes in a single experiment. It will speed up our ability to comprehend pathological processes such as cancer, which include numerous genes and mutations. To find new tumor suppressors or oncogenes, for example, the CRISPR-based genome-wide screening approach can be applied [59]. It opens the door to using CRISPR/Cas9-mediated genome editing to treat or perhaps cure genetic diseases, such as many types of cancer [60].

In genetic engineering, CRISPR/Cas9 offers the opportunity to edit genes, preventing genetic problems from occurring. CRISPR/Cas9 has been explored to target specific oncogenes and tumor suppressor genes to understand and treat cancer. In oncology research, CRISPR/Cas9 targets a tiny fraction of genes. One of the most frequently mutated genes in human cancers is TP53, often called the “guardian of the genome.” It is a critical tumor suppressor gene. To correct TP53 mutations, CRISPR/Cas9 has been used in different models since it can arrest cell cycles, facilitate DNA repair, or promote apoptosis in damaged cells if restored to normal function. Researchers have studied the disease’s development and potential therapeutic interventions with CRISPR/Cas9, targeting mutations in these genes (BRCA1/BRCA2) to increase breast and ovarian cancer risk [49]. An epidermal growth factor receptor (EGFR) mutation has been found in various cancers, including lung cancer, leading to overexpression. Researchers have used CRISPR/Cas9 to knock out EGFR in cell lines to study its role in cancer proliferation and develop drugs that inhibit its activity [49]. A mutation of MYC has been observed in several cancers as it affects cell cycle progression, apoptosis, and cell transformation. To understand the precise mechanisms behind the so-called ‘MYC paradox,’ in which both MYC amplification and suppression contribute to cancer, CRISPR technology has been used to investigate the principle of this paradox [49,61].

These CRISPR/Cas9 approaches are being used for better understanding HCC and as possible therapeutic strategies. Many cases of HCC have specific genetic mutations. For example, the TERT promoter mutation is extremely common in HCC. The CRISPR/Cas9 system is being explored to revert one of the genetic causes of HCC by targeting and correcting these specific mutations within liver cells. Mutations in the CTNNB1 (β-catenin) gene, leading to the activation of the Wnt/β-catenin pathway, are commonly observed in HCC. This pathway is involved in liver regeneration and hepatocarcinogenesis. CRISPR/Cas9 strategies could include knocking out the gene’s mutated forms to prevent uncontrolled cell proliferation. The TP53 gene is often mutated in HCC, leading to the loss of cell cycle regulation and tumor suppression. Cancerous cells can be killed or prevented from expanding by restoring TP53 function via CRISPR/Cas9. Often, HCC involves altered metabolic processes within the liver. IDH (isocitrate dehydrogenase) sometimes changes in HCC, affecting cellular metabolism. By correcting these mutations with CRISPR/Cas9, we can restore normal metabolic function and possibly hinder the growth of cancer cells. The risk of developing hepatitis C (HCV) or hepatitis B (HBV) cancer is higher when infections with these viruses occur frequently. Infections of the liver cells with viral DNA can be prevented from reproducing, reducing the risk of HCC development. CRISPR/Cas9 might be used to disrupt this DNA [25,62].

**Figure 1:**
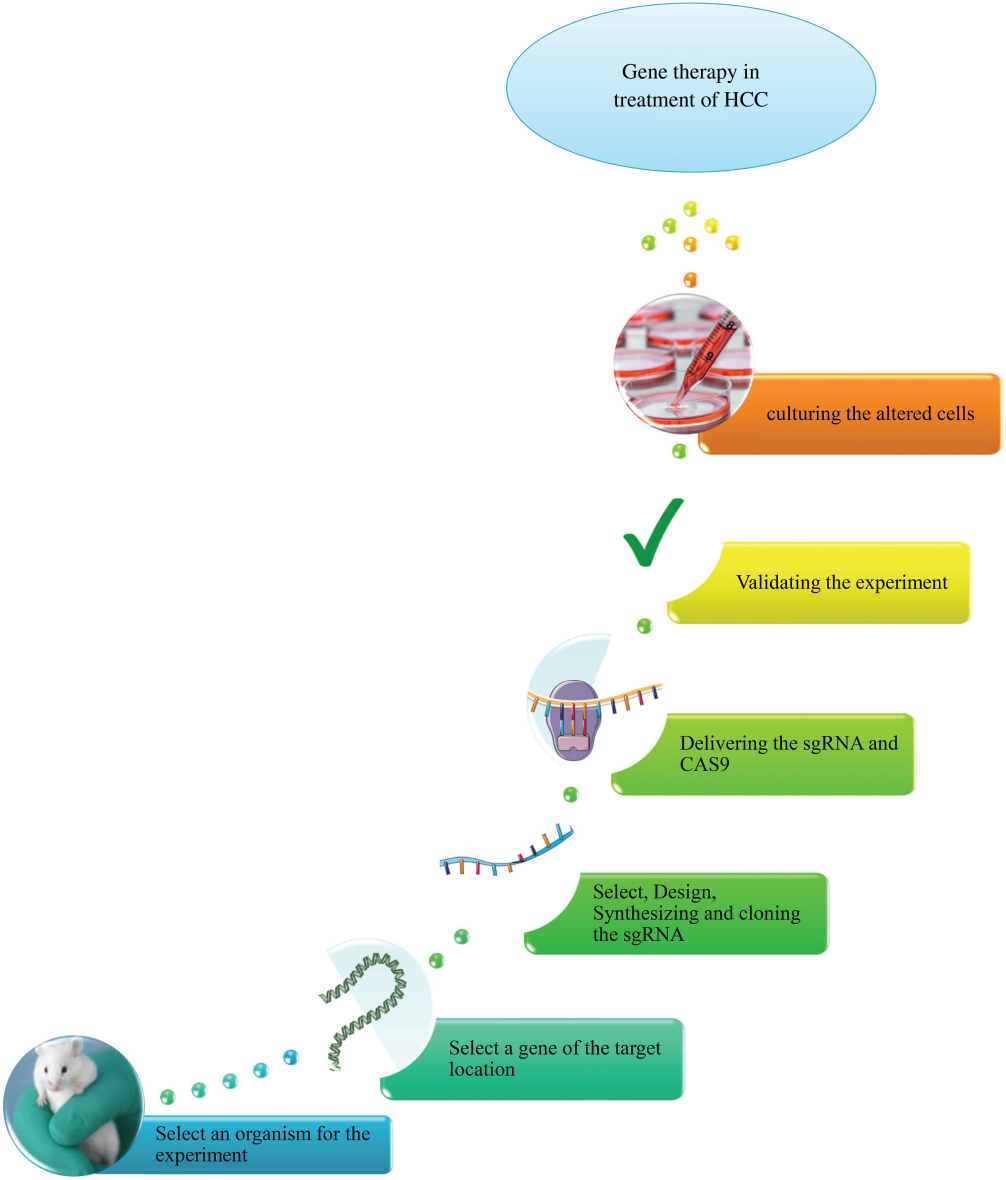
An overview of CRISPR/Cas9 system in gene therapy of HCC.

Taking into account its ever-expanding repertoire of applications, the CRISPR/Cas9 toolkit excels in gene editing and genetic engineering [63]. A simplified approach is illustrated in Fig. 1.

### CRISPR/Cas9 gene editing tool for treating liver cancer

Yang et al. used genome-wide CRISPR/Cas9 knockdown libraries to discover the relevance of pyruvate dehydrogenase β (PDHB), pyruvate carboxylase (PC), and glutamine dehydrogenase α (PDHA). PDHA, PDHB, and PC knockouts disrupt tricarboxylic acid cycling, inhibit mitochondrial function, and inhibit HCC proliferation. In HCC cells, pyruvate was easily absorbed and converted by PDH and PC into acetyl-CoA and oxaloacetate, precursors to citric acid for the TCA cycle. In addition, they demonstrated that pyruvate metabolism could be compensated for during glutamine withdrawal using PDH and PC inhibitors [64].

A metabolic pathway links glycolysis to the citric acid cycle (Krebs cycle) by encoding pyruvate dehydrogenase complex (PDC), which is encoded by PDHA and PDHB (PDC). Acetyl-CoA is made in the Krebs cycle after pyruvate (formed from glucose through glycolysis) is converted into the PDC [65]. It is common to find that cancer cells, including HCC, undergo an altered metabolism known as the Warburg effect. These cancer cells use glycolysis for energy instead of anaerobic glycolysis under normal oxygen conditions (aerobic glycolysis). Cells that proliferate quickly require biosynthesis to maintain their metabolic activity. With CRISPR/Cas9 technology, PDHA and PDHB are disrupted [65], causing pyruvate to build up and acetyl-CoA to be reduced. As a result, the Warburg effect is disrupted, which results in the death of the HCC cells (apoptosis) and decreased energy. The PC enzyme performs its function by converting pyruvate into oxaloacetate. Gluconeogenesis (producing glucose from smaller precursors) depends on oxaloacetate, a crucial intermediate in the citric acid cycle.

Similarly to PDH, PC plays a central role in maintaining gluconeogenesis and citric acid cycle balance. As cells form intermediates of a metabolic pathway through anaplerosis, they may depend on PC. Inhibiting PC will reduce the amount of oxaloacetate, which will disrupt the citric acid cycle and gluconeogenesis. Cell growth and division are slowed due to this disruption by starving cancer cells of energy and other molecular building blocks. Moreover, oxidative stress can result from an imbalance in these pathways, which further induces cell death [65–67].

Hu et al. found that CRISPR/Cas9-mediated gene deletion of Traf3 increased proliferation, migration, and invasion of HepG2 cells, providing valuable insights into Traf3’s function and mechanism [68].

Under conditions of NRAS overexpression, the knockout of PTEN resulted in lipid droplets that resisted metabolic stress, senescence, and immune clearance of premalignant hepatocytes. Consequently, it leads to cell survival and proliferation, which, in turn, leads to cancer. Since liver PTEN is lost somatically and NRAS is overexpressed, the role that hepatocellular lipid droplets play in developing liver cancer cannot be overstated [69].

An exon-skipping variant of Ctnnb1 is produced *in vivo* when a single guide RNA binds to exon 3. Mouse liver tumors were made by Ctnnb1 exon skipping using CRISPR/Cas9 in conjunction with YAPS127A. Oncogene gain-of-function mutations *in vivo* under β-catenin-dependent tumorigenesis are helpful using CRISPR/Cas9 [70].

It is based on these fundamental pathways that TRAF3, PTEN, and CTNNB1 have important roles to play in HCC, as well as their potential anti-tumor effects when these genes are knocked out using CRISPR/Cas9 technology [71–73]. A unique signaling adapter, TRAF3 plays a dual role in innate immunity and various cellular processes, including cell survival. It is capable of negatively regulating the pathways that are involved in the NF-κB signaling pathway [74–76]. According to some researchers, the loss of TRAF3 in some contexts has led to overactivation of NF-κB signaling, which makes the inflammatory response to HCC more proliferative, contributing to the emergence of an environment which has been found to support tumor growth and survival [77–80]. Surprisingly, TRAF3’s role in cancer can be complex. In some contexts, TRAF3 acts as a tumor promoter despite its loss, potentially promoting cancer. It could lead to increased apoptosis and reduced tumor progression by knocking out TRAF3 in those specific contexts. A well-known tumor suppressor gene, PTEN, may significantly affect the tumor’s cellular and molecular environment. As a result of its inhibitory effect on the PI3K/AKT signaling pathway, it is crucial to cell proliferation and survival. HCC, for example, is characterized by unchecked cell growth and survival when PTEN function is lost. In the context of cancer, it is often sought to restore PTEN function rather than knock it out. A CRISPR/Cas9-mediated re-establishment of the PTEN gene could inhibit PI3K/AKT signaling, induce cell cycle arrest, promote apoptosis, inhibit tumor growth, and inhibit tumor growth by correcting loss-of-function mutations in the gene. β-catenin is a major component of the Wnt signaling pathway encoded by CTNNB1. By inactivating CTNNB1, β-catenin accumulates in the cytoplasm and is translocated into the nucleus, stimulating the transcription of genes that promote cell survival and proliferation, contributing to the progression of HCC. In HCC models, CRISPR/Cas9 knocks out CTNNB1 to disrupt the aberrant Wnt/β-catenin signaling pathway, decreasing cell proliferation and increasing death [81–83]. As a result, tumor infection and metastasis could be inhibited as well as the microenvironment surrounding the tumor [84–86].

Cancer cells’ proliferation, migration, and invasion are suppressed by the blockade of the Wnt/β-catenin signaling pathway. Additionally, tumor xenografts were reproducible *in vitro* and *in vivo* in nude mice. By knocking out NSD1, H3, and Wnt10b in HCC cells, they could be considered potential targets for HCC [87].

As a result of efficient loss-of-function oncogene editing by pCas9 delivered by LBP4, it is an effective cancer inhibitor *in vivo* and can increase drug sensitivity. This study demonstrated that LBP/pCas9 complexes enhanced drug antineoplastic effects by causing survivin knockouts in HCC cells by editing their genes and inhibiting their proliferation *in vitro*. Furthermore, pCas9/LBP complexes enhanced the drug antineoplastic effects by inducing survivin knockout [88].

Liu et al. have revealed that transarterial embolization in HCC is more effective with a knockout of HIF-1α mediated by CRISPR/Cas9. It suggests HIF-1α might be a potential clinical knockout target for HCC treatment when combined with transarterial embolization/transarterial chemoembolization. Increasing apoptosis and suppressing cancer cell invasion significantly affected the HIF-1α knockout [89].

The researchers found that metformin is a synthetic lethal target of DOCK1 in HCC, whose levels regulate its antitumor activity. Since metformin activates RAC1 by phosphorylating DOCK1, which then activates DOCK1 to increase cell survival, metformin resistance arises. Patients with metformin-resistant HCC may benefit from combining metformin with inhibition of DOCK1 because the effectiveness of metformin depends on DOCK1 levels [90].

When PTPMT1 is knocked out, cardiolipin does not develop in hypoxia, resulting in electron leakage and reactive oxygen species (ROS) buildup due to improper assembly of the electron transport chain complex. The PTPMT1 inhibitor alexidine dihydrochloride is highly effective compared to HCC cells, especially under hypoxic conditions. The relationship between hypoxia and cancer development suggests that PTPMT1 is a protective factor [91].

Wang et al. demonstrated that cancer CXCR4 modulates cisplatin sensitivity through MDR1. It has also been shown that HepG2 cells that have CXCR4 downregulated have a higher cisplatin tolerance. Through CRISPR/Cas9, the epithelial-mesenchymal transition was reversible, chemosensitivity increased, and cancer malignancy decreased [92].

Lu et al. observed that CRISPR/Cas9 facilitated HCC invasion and migration *in vitro* and *in vivo*. The Keap1/Rhf2/MMP2 signaling pathway may lead to slower and more invasive behavior when SQSTM1/p62 is knocked out. SQSTM1/p62 can be pharmacologically or pathologically regulated to affect migration and invasion of HCC cells. While SQSTM1/p62 has been demonstrated to be an effective target for HCC invasion and migration, further studies are required [93].

Developed in wild-type mice by a single oncogenic modification, this mouse model is the first to generate liver cancer somatically and without toxins or carcinogens [94]. According to immunohistochemical analysis, mouse FL-HCC exhibits hepatocytic and cholangiocytic markers similar to human FL-HCC [95]. It was also reported that fibrolamellar hepatocellular carcinoma (FL-HCC) can be replicated in mice based on phenotypic and genotypic characteristics. In FL-HCC, the recently discovered DNAJB1–PRKACA fusion causes oncogenic transformation and strong pathodiagnosis features. Thus, DNAJB1–PRKACA may be a promising target for FL-HCC treatment, and mice models may be utilized to study FL-HCC formation and progression and develop novel therapeutics [94].

The NFE2L2 gene was precisely knocked down using ultrasound control of CRISPR/Cas9 for tumor-specific gene editing. In contrast to SDT, gene-editing knockdown of NFE2L2 reversed its limitations and increased oxidative stress levels, allowing for a synergistic combination of SDT, gene editing, and antioxidants. This technique is more appropriate for treating deep tumors, such as HCC, since sonodynamic therapy (SDT) penetrates deeper than light-controlled gene editing techniques. In this study, intractable ROS-based SDT issues are addressed in order to design CRISPR/Ca9 delivery systems with promising clinical translation. As a result, this technology can also be applied to other diseases, such as degenerative brain ailments, genetic diseases, and inflammatory disorders. It can also be integrated with immunotherapy to inhibit the growth of metastases and recurrences [96].

CRISPR/Cas9 can also screen genes associated with invasion and metastasis of liver tumors *in vivo* (mouse model). HCC metastases and a better prognosis were related to the expression of XAGE1B and MYADML2, respectively. A significant increase in MYADML2 protein expression was also found in patients over 60 with HCC. MYADML2 levels also increased in HCC, which decreased the cancer’s sensitivity to chemotherapy. Identifying new potential biological targets can improve HCC prognosis and treatment approaches [97].

Crispr-HGF transfection was analyzed in HCC, Huh7, and Hep3B cells to dissect the apoptotic processes involved. Crispr-HGF, when transfected into HCC cells, suppressed the cells’ natural apoptotic response. The conditioned medium of cells treated with Crispr-HGF also showed decreased HGF production. It is noteworthy that Crispr-HGF-transfected cells produced fewer colonies than non-transfected cells. Accordingly, the study’s findings suggest CRISPR/Cas9-mediated regulation of HGF gene expression may play a crucial role in HCC cell apoptosis. The experiments were only conducted at a cellular level, so more research needs to be done to target cancer cells specifically. By knocking out HGF in HCC, researchers can gain insight into apoptosis mechanisms, which could be used to develop anti-cancer therapies [98].

For the first time, PSTK has been identified as a mediator of human cancer cell resistance to ferroptosis. HCC cells were more susceptible to targeted treatments that could induce ferroptosis due to PSTK depletion, suppressing GPX4 activation, GSH metabolism, and folate synthesis. Thus, selenocysteine caused sublethal ferroptosis. It has been demonstrated that Punicalin, a treatment agent for HBV, is synergistic with Sorafenib *in vitro* and *in vivo* for treating HCC. As a result, inhibitors of this protein deserve further clinical evaluation in patients with HCC. To overcome resistance to targeted therapy, HCC patients may benefit from inhibiting PSTK [99].

Many diseases have found success using pigs as big animal models because of their striking parallels to human anatomy, physiology, genetics, and medication metabolism. Autologous liver transplantation has been used to investigate TP53R167H- and KRASG12D-driven HCC. To isolate homogeneous clones of porcine HCC cells, CRISPR/Cas9 was utilized to create genetically customized cells. Through a large animal model and targeting ARID1A in porcine HCC cells, researchers examined how clinically significant mutations affect patients’ propensity to develop cancer and their response to therapy. Because NGS is accurate and sensitive, it was used to analyze CRISPR editing [100].

According to recent research, exon skipping with CRISPR/Cas9 activates intrinsic β-catenin signaling and inhibits immunoactivating cytokines such as CCL20 and CXCL2 to help suppress immune evasion. Approximately 30% of all HCC cases result from mutations of the TNNB1 gene, but ICI therapy cannot be used to treat this subtype. Recombinant cytokines are also being studied as immunostimulants in cancer patients [101], and trans-arterial infusion of the candidate immunoactivating cytokines may also be effective [102].

A functional knockout screen with GeCKO libraries identifies genes involved in the proliferation and metastasis of HCC. ADAMTSL3 and PTEN genes were predicted to proliferate and metastasize by sgRNAs. ADAMTSL3 is involved in HCC proliferation and metastasis, and future studies will examine these genes. When ADAMTSL3 is removed from HCC cells, it can proliferate. In HCC, loss of function of ADAMTSL3 was found to be associated with proliferation for the first time. Our results further demonstrate that PTEN loss-of-function inhibits HCC cell invasion, proving the effectiveness of CRISPR-based *in vivo* screening despite showing that PTEN loss-of-function leads to tumor dissemination and invasion [103].

In a first-of-its-kind study, Song et al. generated sgRNA containing a specific sequence and used CRISPR/Cas9 to create HCC cells expressing HBsAg knockout. HBV-HCC’s malignant potential can be increased by HBsAg through gain- and loss-of-function mechanisms, as shown by their findings *in vitro* and *in vivo*. IL-6/STAT3 and STAT3 may mediate HBV-HCC’s HBsAg-mediated signaling pathways. As demonstrated here, CRISPR/Cas9 technology targets the ORF of HBsAg, thereby providing a novel therapy for HBV-related HCC [104].

Based on Saputra and colleagues’ model, it appears that non-synergistic (anti-synergistic or additive) drug combinations are more effective at suppressing the spread of resistance subclones than synergistic ones and provide long-term protection against resistance [105].

It has been shown that porcine HCC cells that are KOed for AXIN1 and/or ARID1A are less susceptible to sorafenib and doxorubicin by CRISPR KO. Moreover, the study suggests that pigs may be injected with autologous cells to create genetically tailored tumors. A CRISPR-edited cell injected intrahepatic will allow additional genetically tailored HCC models based on the Oncopig model to be developed. Developing precise porcine HCC models would be possible through *in vivo* editing of HCC driver genes in porcine hepatocytes using CRISPR components. In addition to these translational HCC models, innovative precision medicine can be tested, and effective therapeutics can be identified to treat frequently occurring gene mutations using these promising tools [106].

CRISPR genome-wide screening found that NCAPG expression significantly predicts tumor recurrence in the non-structural maintenance of chromosomes. Surgical resections and liver transplants are the best treatment options for HCC. Based on an AUC of 0.80 in 2 separate datasets, the authors claim that NCAPG transcript levels and liver cirrhosis may be utilized to distinguish early recurrent cancers from non-recurrent tumors. The protein level of NCAPG can accurately identify seven out of eight early recurrent tumors. Studies have also demonstrated that patients with high levels of NCAPG have a significantly reduced chance of surviving disease-free [107].

With CRISPR/Cas9, the tyrosine kinase Axl receptor was knocked out in human hepatoma cell lines HLF and SNU449. Validation of HLF-Axl-1, HLF-Axl-2, SNU449-Axl-1, and SNU449-Axl-2 cells has been completed using two single clones of genomic editing events (HLF-Axl-1, HLF-Axl-2). As a result of ELISA analysis, both total Axl and soluble Axl were detected in the supernatant of incompletely edited SNU449-Axl--1 cells. However, immunoblotting did not reveal the expression of the Axl protein. As a result of these experiments, it appears that genomic heterogeneity may contribute to incomplete editing with CRISPR/Cas9 in cancer cells [108].

Human Huh-7 and HepG2 cell lines were treated with CRISPR/Cas9 to eliminate the binding sites of the MT family, and compared to CRL-12461, a normal liver cell line, Huh-7 and HepG2 cells generated much more MT transcription. Moreover, changes in H3K4me3 and H3K9me3 levels of the MT gene were found after CTCF binding domain destruction after comparing the CTCF binding area with the CTCF binding domain in both the chromosomal conformation capture technique (3C) and the chromatin immunoprecipitation technique (ChIP). The change in local genomic organization to regulate gene transcription was investigated as a potential disease treatment method [109].

These studies demonstrate how gene editing with CRISPR/Cas9 can be used in experimental models to produce specific, measurable effects against HCC pathogenesis. Their work highlights how molecular characteristics play a critical role in determining cancer behavior and treatment strategies, illustrating the therapeutic potential of targeted genetic interventions.

HCC is treatable by CRISPR/Cas9 in both *in vitro* and *in vivo* studies. These studies require a better understanding of HCC’s potential mechanisms and therapeutic targets. Clinical trials must also be conducted on human samples to translate these findings into clinical practice. HCC therapy using CRISPR/Cas9 is still in its infancy in clinical trials. It depends on several factors, including safety and efficacy, delivery mechanisms, regulatory and ethical considerations, and individual differences.

### CRISPR/Cas9 and genes related to drug resistance

As part of their screening of genome-wide CRISPR/Cas9 libraries using three sgRNAs and two additional sgRNAs, Wei et al. found that PHGDH contributes to resistance to Sorafenib. In addition to PHGDH, AKT1S1, TBL1Y, SKAP2, and AMPD2 were also found to contribute to resistance to Sorafenib. Previously, pooled shRNA knockdown library screenings in mice also revealed that MAPK caused HCC resistance to Sorafenib. When Sorafenib is administered to HCC cells, PHGDH inactivation leads to increased apoptosis. Inactivation of PHGDH increases cell death in HCC cells [110].

A critical issue for HCC research is the development of sorafenib-resistant cancer models. PHGDH, an enzyme responsible for serine biosynthesis, a component of nucleotide and protein synthesis, plays a major role in this process. A tumor’s metabolism is often altered, and for rapid cell division, it requires an increased supply of serine. PHGDH catalyzes the first step of serine synthesis, and the enzyme controls its flux. In addition to producing serine also contributes to DNA and RNA replication, which is essential to cell proliferation by supporting nucleotide synthesis. PHGDH is knocked out with CRISPR technology, disrupting the serine synthesis pathway. Several intracellular consequences result from this, including reduced nucleotide availability, oxidative stress, DNA damage, and impaired antioxidant defense, which increases sorafenib sensitivity. It becomes obvious why PHGDH knockout would increase HCC cells’ susceptibility to sorafenib-induced apoptosis by linking its role in cell survival and proliferation with the stress and DNA damage response [111–113].

CRISPR activity in hepatoma cells led to the overexpression of genes that conferred resistance to regorafenib. Hepatoma cells whose HK1 gene has been mutated resist the chemotherapy drug regorafenib. It has been observed that glycolysis inhibitors can be made more effective using pharmacological means, such as lonidamine, which resensitizes cells to regorafenib. The FDA has not yet approved such inhibitors. Further, HK1 expression is ubiquitous, so inhibition of other identified genes would be useful once further validated, as it is unlikely that HK1 can be used to treat patients resistant to TKIs. By assessing the expression levels of HK1 or one of the other targets, it may also be possible to predict the outcome of TKI therapy, thus eliminating unnecessary morbidity and costs associated with inadequate candidates [114].

As part of glycolysis, HK1 is crucial for converting glucose into glucose-6-phosphate, the first step. As a result, cells produce energy and metabolites required for various cellular functions, especially those that need high energy. Below are details about how HK1 mutations can result in regorafenib resistance. Cancer cells rely more on glycolysis for energy production despite aerobic conditions, known as the Warburg effect. HK1 plays an important role in this process. In the presence of regorafenib, which typically inhibits tumor proliferation and angiogenesis, a mutation in HK1 could alter the enzyme’s activity, making cells more energy-producing and able to survive. Cancer cells require glycolysis to survive, and many have mechanisms to maintain it even when stressed. These cells might resist regorafenib’s effects if HK1 mutations make them less dependent on the metabolic pathways that regorafenib targets. Regorafenib triggers apoptosis in cancer cells so that they may develop a workaround to the drug-induced metabolic disruption. For these cells to survive, they need a functioning glycolytic pathway, which provides them with the energy they need to avoid apoptosis. By avoiding the usual pathways leading to programmed cell death, HK1 mutant cancer cells might develop resistance to the drug if they can continue metabolizing glucose efficiently. It works by targeting multiple kinases involved in cancer growth and progression. The mutant form of HK1 can cause changes in cellular signaling pathways. It can cause the drug to fail to work as intended, as the cells ‘ignore’ the drug’s effects [115].

The main obstacle to the successful treatment of advanced liver cancer is drug resistance to lenvatinib. A recent research located the key gene linked with Lenvatinib resistance in HCC with the sole screen of the CRISPR/Cas9 genome library. This gene was found to be DUSP4, which may serve as an important guide to improve resistance to inhibitors related to tyrosine kinases in the future. In patients with HCC who lack DUSP4, Lenvatinib reactivates ERK and MEK. *In vivo* experiments successfully used a xenograft mouse model [116].

When expression of DUSP4 is maintained at normal levels, it prevents excessive proliferation and tumor formation by inactivating the MAPK pathway, including ERK1/2. By reducing or losing DUSP4, ERK1/2 is dephosphorylated less, which leads to its phosphorylation, causing it to activate. As a result of ERK activation, genes involved in cell survival, growth, and proliferation can be transcribed into the nucleus, increasing cell resistance to drugs such as lenvatinib’s antiproliferative effects. Several tyrosine kinase receptors are frequently upregulated in cancers, and lenvatinib inhibits them by inhibiting them. Cancer cells can survive and proliferate by triggering these receptors, including the MAPK/ERK pathway. Even if lenvatinib inhibits upstream receptors when DUSP4 is lost, the ERK pathway remains active or hyperactive, negating its effect on slowing tumor growth. Researchers can strategize how to target ERK signaling if they understand that the loss of DUSP4 results in ERK signaling reactivation. Lenvatinib treatment could be combined with additional inhibitors of the ERK1/2 pathway directly (often referred to as MEK inhibitors). By shutting down the survival signals that cancer cells rely on due to the loss of DUSP4, this combination therapy could overcome resistance [117].

A study by Chen et al. found that knocking down the KEAP1 gene could prevent sorafenib’s resistance to NRF2 and investigated whether an inhibitor of NRF2 could stop HCC growth in synergy with sorafenib. Further, they discovered that FGF21 has a crucial role in downstream NRF2 regulation. A positive feedback loop resulted from FGF21 binding to NRF2 through its C-terminus, which reduced NRF2’s ubiquitination and stabilized the protein. In light of these findings, inhibiting FGF21 may be useful for managing sorafenib resistance in HCC [118].

Genome-wide CRISPR transcriptional activation libraries (SAM) were used as part of the first study of sorafenib resistance. Compared to the previous research screened for sorafenib resistance with GeCKO v.2 [119], researchers have made impressive and different discoveries by combining gene expression profiling data of Huh7 cells with the SAM library. HCC cells can develop resistance to sorafenib as a result of LRP8, according to research. It was found that all three sgRNAs targeting LRP8 were present in Huh7 cells with high copy numbers. The expression of LRP8 in HCC cells exposed to sorafenib was also significantly increased. The overexpression of LRP8 in HCC cells was also associated with decreased apoptosis and increased β-catenin activity [120].

Through research, new information has been collected that has helped to clarify why lenvatinib resistance occurs in HCC. Hypomethylation and driver mutations, such as TP53, both cause up-regulation of LAPTM5. Lenvatinib and hydroxychloroquine (HCQ) inhibit autolysosome formation, which inhibits tumor growth. By knocking down LAPTM5 or injecting HCQ, autophagy inhibition can yield promising results to overcome resistance and improve patient survival by combining autophagy inhibition with Lenvatinib [121].

The tumors were treated with regorafenib or a vehicle based on CRISPR kinome libraries, which produced Cas9-expressing HCC cells for xenograft studies. According to the sequence analysis, regorafenib-treated tumors have 31 more abundant genes than vehicles-treated tumors, including two paralogues of LATS2, which is an integral part of the Hippo signaling pathway. YAP or Bcl-xL inhibition of regorafenib-insensitive HCC cells made them regorafenib-sensitive. Based on screening for CRISPR loss-of-function in HCC, regorafenib is effective against HCC [122].

Repurposing and selecting the best effective treatment choices by examining current medications and combinations is intriguing. A CRISPR/Cas9 screening study has indicated that ifenprodil and sorafenib combine to treat HCC effectively. A combined therapy regimen of IFEN and SOR might be an effective strategy for SOR-resistant cancer cells. This combination may not adequately inhibit the proliferation of cancer cells that have become resistant to treatment [123].

As shown in a study, CRISPR screens can help identify therapeutic targets for sorafenib treatments. SGOL1 expression is a prognostic indicator for sorafenib treatment with NGS combined with CRISPR/Cas9 genome editing. The expression of SGOL1 in HCC cells with high differential expression may indicate a poor prognosis. Thus, treating HCC patients with sorafenib and SGOL1 could prolong their lives by months or even years [119].

Compared with control cells, HUH-7 cell viability was enhanced when treated with a modest dosage of sorafenib or lenvatinib. As we report, KEAP1 is inactivated, which has important clinical implications. KEAP1 was the best candidate gene after undergoing a process called Model-Based Analysis of Genome-wide CRISPR/Cas9 Knockout, or MAGeCK for short. In both short- and long-term treatments with sorafenib, KEAP1-disrupted HCC cells showed less sensitivity. KEAP1-disrupted cells displayed reduced sorafenib-induced ROS than wild-type cells. Several recent HCC treatments, including regorafenib, have also demonstrated resistance to cells disrupted by KEAP1 [124].

Recently, data suggest that sorafenib enhances the therapeutic effect of IQGAP1 and FOXM1 by preventing crucial signaling pathways such as PI3K/AKT and MAPK/ERK by knocking down IQGAP1 and FOXM1, in combination with sorafenib administration. Secondly, CD133+ cancer stem cells were impaired, which plays a key role in sorafenib resistance. Sorafenib and EVs may be combined in future anticancer treatments to provide improved outcomes over solely using sorafenib [125].

### CRISPR/Cas9 and lncRNAs in HCC

In the study of SNHG9, CRISPR-dCas9 was used to investigate its biological function. They found that reducing SNHG9 expression inhibited HCC metastasis and investigated the molecular mechanisms responsible for it. SNHG9 is responsible for gene expression, tumor genesis, and DNA methyltransferase binding, so its knockout might demethylate the GSTP1 promoter [126].

An analysis of the long noncoding RNA (lncRNA) SNHG9 (Small Nucleolar RNA Host Gene 9) in HCC indicates that it has an important role to play in tumor progression and metastasis [127]. It is well known that lncRNAs play a crucial role in regulating various cell processes, including cancer progression. They may affect transcription, mRNA stability, or translation without coding for proteins, thereby modulating gene expression. CRISPR technology might disrupt the cell cycle progression by knocking out SNHG9, possibly through the regulation of genes related to cell cycle progression, which is essential for the proliferation of cancer cells. A loss of SNHG9 could reduce cancer cell proliferation, reducing tumor growth and metastasis opportunities. These processes are crucial for metastasis, enabling cancer cells to move and invade surrounding tissues and, eventually, distant organs. In addition to interacting with or regulating the expression of proteins involved in cytoskeleton rearrangement and cell adhesion, SNHG9 is also involved in cytoskeleton rearrangement [127]. A knockout of SNHG9 could impair migration and invasion, decreasing the ability of HCC cells to spread metastatically. A knockout of SNHG9 could cause programmed cell death unintentionally. The absence of SNHG9 would remove this apoptosis inhibitor, making cancer cells more susceptible to external death signals and anti-cancer therapies, resulting in smaller tumors and fewer metastatic spreads. The expression of SNHG9 may be directly related to metastasis-related genes. A cell survival signaling pathway that promotes cell survival in distant tissues might be modulated by it, such as matrix metalloproteinases (MMPs), which are involved in tissue remodeling and invasion. Its knockout would disrupt these processes, reducing the ability of cancer cells to spread. Cancer cells must be able to enter the bloodstream to spread to other organs, and angiogenesis, which is responsible for forming new blood vessels, is necessary for this process. If SNHG9 acts as an angiogenesis inhibitor, its knockout would impede the vascularization that supports tumor growth and the access of cancer cells to the bloodstream [128].

The groups differed significantly in miRNA-4764-5p and lncRNARP11-156p1.3 expression. A significant reduction in viability and cell count was observed in HEPG2 cells knocked out with CRISPR/Cas9 mediated knockdown of RP11-156p1.3. The selected RNAs may serve as potential therapeutic targets for HCC and are important pathogenic players [128].

In most cases, researchers conduct cell viability assays (such as MTT or MTS assays) after RP11-156P1.3 is knocked out in HCC cells [129,130]. As a result of RP11-156P1.3 knockout in HCC cells, they may report a significant drop in cell viability, quantified by a percentage. For example, “The viability of HCC cells was reduced by 40% compared to control cells 72 h after knockout.” One could determine the effect on cell proliferation using an assay such as BrdU or cell counting. The results could be expressed as a percentage of proliferation or as a percentage of the number of cells at different time points. After four days, cell proliferation was significantly reduced by 35% following RP11-156P1.3 knockout. A colony formation assay could demonstrate a decreased oncogenic potential by measuring how many colony forms from CRISPR-edited cells compared to control cells. Following the deletion of RP11-156P1.3, HCC cells showed a marked reduction of 50% in the number of colonies established over two weeks. Researchers used flow cytometry to analyze cell cycle data to determine whether the knockout of lncRNA led to an increase in G1 cells, indicating cell cycle arrest. The cell cycle distribution changed significantly with RP11-156P1.3 knockouts, with a 25% increase in G1 cells, suggesting RP11-156P1.3 regulates the cell cycle”. The final step would be to stain and analyze cells using Annexin V/PI, followed by flow cytometry. An increase in early and late apoptotic cells could provide a compelling indication that RP11-156P1.3 contributes to cell survival. A pronounced apoptotic response was triggered by the knockout of RP11-156P1.3, resulting in an increase of 30% in Annexin V-positive cells [131–134].

It has been shown that genome-wide CRISPR activation screening is an effective method of identifying oncogenic lncRNAs, which can then be targeted clinically. The authors comprehensively showed that lncRNAs have a physiological role in driving HCC development by computational analysis of clinical transcriptome data sources and functional CRISPR activation screening. As demonstrated by CASC11’s ability to regulate MYC and its downstream targets, HCC progression is regulated. Hence, functional lncRNA candidates must be further investigated to translate into biomarkers and therapeutic targets for patients with HCC [135].

As a result of CASC11, MYC expression is regulated through a specific pathway. For example, CASC11 could boost Wnt/β-catenin signaling pathways, which are crucial for cell survival and proliferation. By preventing its degradation, CASC11 may increase β-catenin’s nuclear translocation within this pathway, stabilizing β-catenin. β-catenin promotes the transcription of MYC and other target genes after binding to the TCF/LEF family of transcription factors. Cell cycle progression, inhibition of apoptosis, and enhanced cellular metabolism are all integral to the aggressive phenotype of cancer cells, which are all facilitated by this upregulation. Researchers would probably measure CASC11’s effect on HCC progression quantitatively to understand the extent of its influence. For example, a CRISPR/Cas9 knockout of CASC11 in HCC cell lines could be observed, and then tumor properties might be altered accordingly. A significant decrease in cell proliferation was observed through cell proliferation assays (such as MTT/MTS); for instance, “CASC11 knockouts resulted in a 60% reduction in cell proliferation after 72 h in comparison to control cells.” Migration and invasion assays (such as wound-healing and transwell assays) might also suggest a substantial decline in metastatic properties. An analysis of qRT-PCR and Western blots would be used to assess MYC levels post-CASC11 knockout, showing, for example, an “80% reduction in MYC mRNA and 90% reduction in MYC protein”. It is also possible to demonstrate using mouse models that CASC11 knockout decreases tumor growth in a significant way. Researchers may state, “Tumors in the CASC11 knockout group were 50% smaller than those in the control group”.

Several considerations must be considered as lncRNAs such as SNHG9, RP11-156P1.3, and CASC11 in HCC transition from laboratory to clinical settings. Several cancer types, including HCC, have expressed lncRNAs in a cell-specific manner, making them possible as prognostic markers. A researcher might examine the correlation between SNHG9, RP11-156P1.3, and CASC11 levels and the disease progression, patient survival, and therapy response. LncRNAs can be predictive biomarkers if they correlate strongly since some lncRNAs are involved in oncogenesis, metastasis, and resistance to therapy. If these lncRNAs are important for HCC, they may be targeted as treatments. These lncRNAs might be knocked down by CRISPR/Cas9, potentially impairing the progression of cancer cells rather than just being used for research. Developing lncRNAs that can be used as prognostic markers or therapeutic targets requires clinical trials, in which the safety and efficacy of targeting these lncRNAs in humans will be assessed. In preparation for phased clinical trials, preclinical studies are often performed on cell lines and animal models first to evaluate safety (Phase I) and then efficacy (Phases II and III) [136,137].

### CRISPR/Cas9 and nanoparticle agents

With the use of Hep@PGEA, a therapy vector for orthotopic HCC that can reverse its charge has been successfully produced. By increasing pCas9 release and releasing it for charge reversal using keys, Hep@PGEA can effectively condense and deliver pCas9 to orthotopic HCC. The proposed therapeutic strategy did not appear to have any apparent toxicity in HCC [138]. It seems that gene therapy combined with sorafenib and gene therapy combined with Hep@PGEA/pCas9 complexes is an effective way of treating HCC, providing a novel therapeutic approach. Further, due to their high liver enrichment, Hep@PGEA-based delivery systems could also be used to treat other liver diseases [139].

As a result of optimizing the nanosystem, the drug and gene payloads were efficiently loaded and released, achieving a loading efficiency of 23.15 percent for sorafenib and 76.65% for pEGFR. According to the *in vitro* release study of SEHPA NPs, they release 2-fold more at pH 5.5 than at pH 7.4 over 72 h. At 24 h after injection, SEHPA NPs were 2-fold more abundant in tumor tissues in tumor-bearing mice than unmodified nanoparticles. At 16 days post-treatment, SEHPA NPs achieved 85% tumor growth inhibition *vs*. 52% with sorafenib alone due to their improved tumor targeting and synergistic therapy. Further, SEHPA NPs reduced tumor weights by 62% at the study end, compared with just 21% for sorafenib alone. It was able to deliver and combat tumors efficiently due to the rational design of the nanosystem.

As part of a study using hollow mesoporous silica nanoparticles coated with polyamidoamine-aptamers, sorafenib and CRISPR/Cas9 co-delivery were demonstrated. As a result of the core-shell nanoparticles’ exceptional stability, the gene-drug concoction was successfully delivered, with a substantial amount of drug placed inside the particles and their release carefully regulated. SEHPA nanoparticles were found to have a stable release profile, a long half-life in the blood, and a decent circulation time. Although neither HE staining nor blood biochemical tests could identify SEHPA NPs, they accumulated in the tumor area. After EGFR gene editing *in vivo*, IHC was used to verify inhibited EGFR expression in tumor tissues, and sequencing *in vivo* was used to confirm that gene editing of EGFR was effective. Based on these findings, the nanosystem could be used as a synergistic system for treating HCC by combining gene therapy and chemotherapy [140].

Xenografts were created by injecting human HCC cells engineered to express luciferase into 6-week-old female BALB/c nude mice. We gave this xenograft seven days to establish in the liver parenchyma before starting treatments with fragments from this xenograft. Bioluminescence imaging was used to monitor tumor growth after injecting luciferin substrate, with radiant efficiency based on photons/second/cm^2^/steradians. Apoptosis, bioluminescence radiant efficiency, overall survival, tumor weight, survivin gene editing frequency, survivin protein expression by immunohistochemistry, and survivin gene editing frequency were some of the efficacy endpoints examined. Radiant efficiency was reduced by 60%–70% with the Hep@PGEA/pCas9 treatment, while the combination with sorafenib reduced it by 95%–98%. Overall survival was not different during the 37-day experiment. It was found that the survivin gene editing frequency was 0.0% (control), 0.42g (Hep@PGEA/pCas9), 0.12g (sorafenib), and 0.026g (combination). The apoptosis rate was increased 2-fold with Hep@PGEA/pCas9 and 4-fold with the combination treatment, as opposed to the control treatment. Survivin protein expression was decreased by 60% with Hep@PGEA/pCas9 and 90% with the combination.

The nanosystem has promising results, but several hurdles remain before clinical translation. The work is still in the early stages and has not yet been evaluated in human clinical trials, so demonstrating therapeutic efficacy and safety has been challenging. Comparing the nanosystem with mouse models would provide information on biodistribution, tumor accumulation, gene editing efficiency, and antitumor effects. It is not known whether it is toxic, off-target, or immunogenic, so Phase I trials need to be conducted in humans. Scaling up manufacturing processes and maintaining quality control for clinical good manufacturing practice still pose challenges. Regulatory approval for gene therapy and targeted drug delivery will likely be more complex. Optimizing and testing this nanosystem extensively will be necessary to determine whether it can be translated into human liver cancer patients.

Despite the orthotopic mouse model demonstrating proof of concept efficacy in patients with HCC, further optimization is needed. Tests will be conducted in immunodeficient models, with repeat doses, improved delivery efficiency, on-target editing, scaling up nanoparticle production, testing against survivin sequences, combining with immunotherapy, repeat doses, and phased clinical trials. Although early data is encouraging, more work is needed to address limitations in models, effective delivery, efficient manufacturing, combinatorial strategies, pharmacology, and clinical trials.

**Figure 2:**
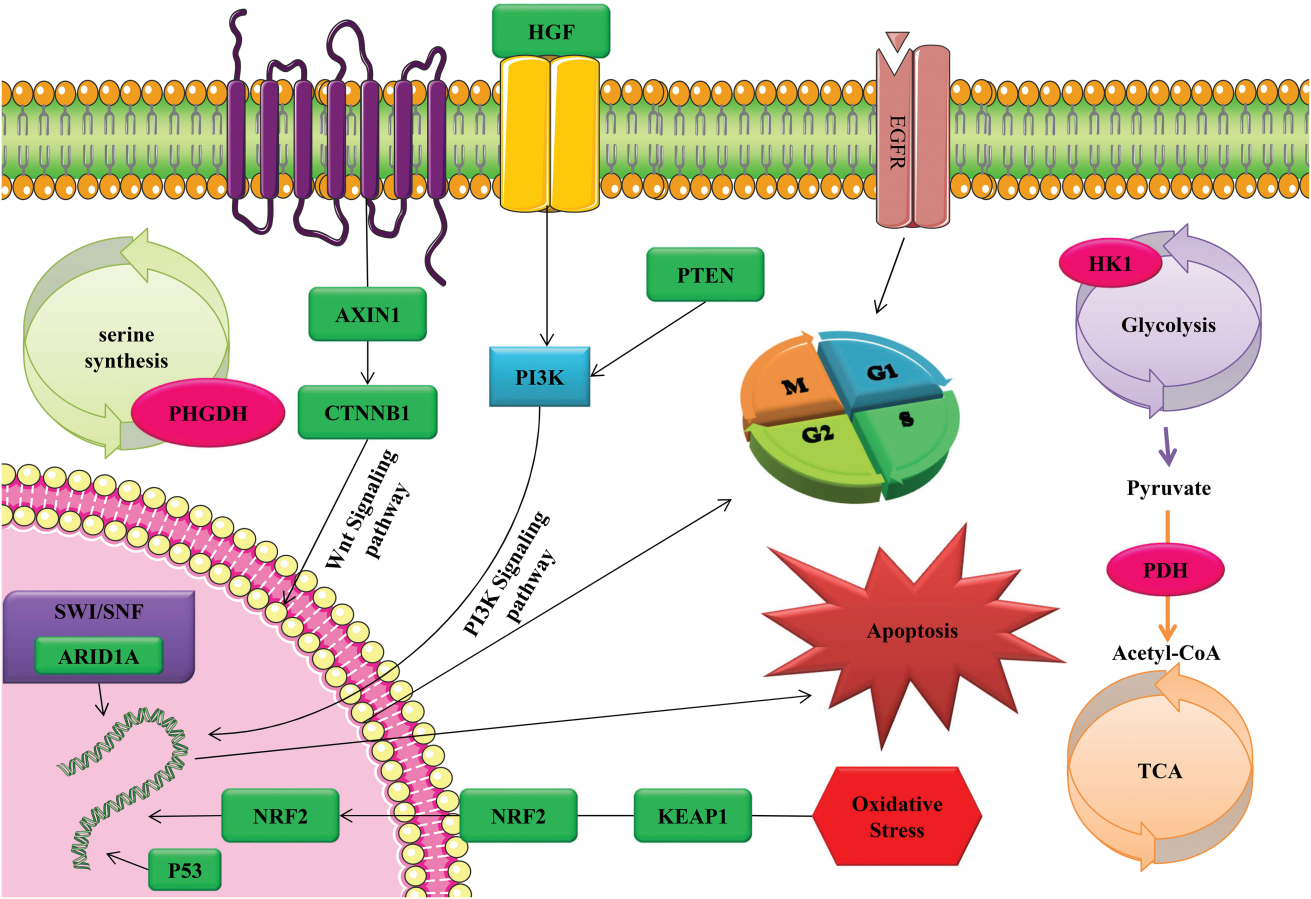
The activation of tumor cell apoptosis, cell cycle, and oxidative stress through CRISPR/Cas9 technology as a gene therapy tool through diverse range of signaling pathways and functional mechanisms.

Finally, all the information and data presented above are shown and listed in Fig. 2 and Table 1. Table 2 provides a simplified view, and it is important to note that the actual scenarios can be much more complex, depending on the specifics of the therapy, individual patient variation, and ongoing advancements in each field. Each approach is subject to extensive research and clinical trials to validate its efficacy and safety, especially in a delicate context like HCC treatment.

**Table 1:** A summary list on CRISPR/Cas9 system for treating HCC including involved tissues/cell lines, genes/targets and their functions

Ref.	*In vivo/In vitro*	Tissue or cell type	Gene/target symbol	Gene/target name	Function
[110]	*In vivo/In vitro*	Mice	PHGDH	Phosphoglycerate Dehydrogenase	Antioxidant
		MHCC97L			Inactivation of PHGDH induced cell apoptosis
	*In vivo/In vitro*	Mice	PDHA	Pyruvate Dehydrogenase α	Pyruvate metabolism
		MHCC97L	PDHB		PDH or PC inhibitors disrupt the TCA cycle induced by dietary glutamine depletion
				Pyruvate Dehydrogenase β	
			PC		
				Pyruvate Carboxylase	
[64]	*In vivo/In vitro*	PDX mice	HK1	Hexokinase 1	Glycolysis-related
		Huh7			HK1 knockdown reduces cell viability in regorafenib-treated cells
		JHH-6			
		HLF			
	*In vitro*	HepG2	SNHG9	Small Nucleolar RNA Host Gene 9	Knockdown of SNHG9 inhibit cell proliferation, block cell cycle progression, and inhibit cell migration and invasion by upregulating GSTP1
		Huh7	(lncRNA)		
			GSTP1		
				Glutathione S-Transferase P1	
[114]	*In vitro*	HepG2	Traf3	TNF Receptor Associated Factor 3	Enhanced the proliferation and invasion ability in the Traf3 knockout group
	*In vivo*	Mice	PTEN	Phosphatase and Tensin Homolog	PTEN knock-out and NRAS knock-in induces HCC and hepatic lipid accumulation
			NRAS		
				Neuroblastoma RAS Oncogene Homolog	
[126]	*In vivo/In vitro*	Mice	CTNNB1	Catenin Beta 1	CTNNB1 exon 3 induces nuclear accumulation of β-catenin
		KPC			
	*In vivo/In vitro*	HCC tissues	NSD1	Nuclear Receptor Binding SET Domain Protein 1	Knockout of NSD1 inhibited the proliferation, migration and invasion abilities
		HL-7702			
		Huh7			
		Hep3B			
		SMMC-7721			
		HepG2			
		SK-Hep1			
[68]	*In vivo/In vitro*	Mice	DUSP4	Dual Specificity Phosphatase 4	DUSP4 knockout enhanced HCC cell survival, cell proliferation and migration during Lenvatinib treatment by regulation of p-ERK and p-MEK levels
		LO2			
		LM3			
		HepG2 Huh7			
		HEK293T			
	*In vivo/In vitro*	Mice	survivin	survivin oncogene	Knockout survivin oncogene produces efficient anti-cancer activities
		BEL7402			
		HEK293			
[69]	*In vivo/In vitro*	Mice	HIF-1α	Hypoxia Inducible Factor-1α	Enhances the antitumor effect of transarterial embolization
		SMMC-7721			
	*In vivo/In vitro*	Mice	DOCK1	Dedicator Of Cytokinesis 1	Inhibition of tumor progression
		PLC			
		Huh7			
		HEK293T Hep3B			
		SNU423			
		SNU449 SNU475			
		CLC1			
		CLC11			
		CLC50			
[70]	*In vivo/In vitro*	Mice	PTPMT1	Protein-Tyrosine Phosphatase Mitochondrial 1	Hypoxic survival and cancer development.
		MHCC97L Hep3B Huh7 HepG2			
		Hepa1-6			
		ES-2			
		HCT116			
		MDA-MB-231			
		PC3			
	*In vivo/ In vitro*	Mice	CXCR4	C-X-C Motif Chemokine Receptor 4	Decreases the malignancy
		HepG2			
[87]	*In vivo/In vitro*	HepG2	NRF2	Nuclear Factor Like 2	Abolish HCC’s growth
		LM3	FGF21		Inducing sorafenib resistance
				Fibroblast Growth Factor 21	
	*In vivo/In vitro*	Mice	SQSTM1/p62	Sequestosome 1	Inhibits migration and invasion
		HEK293T			
		HepG2			
		THP-1			
		SQSTM1			
[116]	*In vivo/In vitro*	HCC patient	RP11-156p1.3	RP11-156p1.3	Knockout of lncRNA- RP11-156p1.3 results in overexpression of miRNA-4764-5p_1 leading to inhibition of TNF-alpha, NF-κB and RFTN1 expression
		HepG2			
			(lncRNA)		
	*In vivo/In vitro*	Mice	DNAJB1–PRKACA	DnaJ Heat Shock Protein Family (Hsp40) Member B1	DNAJB1–PRKACA fusion is responsible for oncogenic transformation and major pathodiagnostic features
		Neuro-2a			
				Protein Kinase CAMP-Activated Catalytic Subunit Alpha	
[88]	*In vivo/In vitro*	Mice	NFE2L2	NFE2 Like BZIP Transcription Factor 2	NFE2L2 gene-editing knock down reversed the limitations of SDT and amplified cellular oxidative stress levels
		hep3B2.1-7			
		HepG2			
	*In vivo/In vitro*	Mice	LMO1 MYADML2	LIM Domain Only 1	High expression of MYADML2
		HEPG2 Huh7			reduced the sensitivity to chemotherapeutic drugs
			PLK4 XAGE1B	Myeloid Associated Differentiation Marker Like 2	
				Polo Like Kinase 4	
				X Antigen Family Member 1B	
[89]	*In vitro*	Huh7 Hep3B	HGF	Hepatocyte Growth Factor	Suppressed cell proliferation and induced apoptosis
	*In vitro*	SK-HEP-1	LRP8	LDL Receptor Related Protein 8	LRP8 has role in Sorafenib resistance
		HEPG2 HEK293T Huh7			
		MHCC-97H			
[90]	*In vivo/In vitro*	Mice	LAPTM5	Lysosomal Protein Transmembrane 5	Induces lenvatinib resistance
		HCC-LM3			
		MHCC97-H			
		MHCC97-L			
		Hepa1–6			
		Huh7			
		HepG2			
		Huh1			
		SK-HEP-1			
		SNU-182			
		SNU-387			
		SNU-423			
		SNU-398			
		JHH-1			
		JHH-4			
		JHH-6			
		JHH-7			
		SNU-449			
		CLC13			
		CLC16			
		CLC25			
		CLC41			
		CLC47			
		CLC50			
	*In vivo/In vitro*	Mice	PSTK	Phosphoseryl-TRNA Kinase	Regulator of chemotherapy-induced ferroptosis
		Hep3B Huh7			
[91]	*In vivo/In vitro*	Mice	survivin ki-67	survivin	Tumor proliferation marker
		BEL7402		ki-67	
	*In vivo/In vitro*	Transgenic pigs (*Sus scrofa*)	TP53	Tumor Protein P53	Tumor progression
			KRAS		
				KRAS Proto-Oncogene, GTPase	
		HCC cell lines			
[92]	*In vivo/In vitro*	Mice	CTNNB1	β‑catenin	Immune evasion
		HuH7			
		3H3			
	*In vivo/In vitro*	Mice	ADAMTSL3	ADAMTS Like 3	Suppressors of HCC proliferation and metastasis
		Hep3B			
		SMMC7721	PTEN		
				Phosphatase and Tensin Homolog	
[118]	*In vivo/In vitro*	Mice	LATS2	Large Tumor Suppressor Kinase 2	LATS2 knockdown mitigated the cytotoxic and proapoptotic effects of regorafenib
		HLF			
		Hep3B Huh7			
	*In vivo/In vitro*	Mice	HBsAg	HBsAg	HBsAg inhibits proliferation and tumorigenicity
		PLC/PRF/5HepG2-.15,			
		Hep3B			
		SK-hep1			
		HLF,			
		Huh-7			
		HEK293F			
[93]	*In vivo/In vitro*	Mice	NMDAR1	N-Methyl-D-Aspartate Receptor Subunit NR1	Cell-cycle arrest
		HepG2 Hep3B	NMDAR2B		Downregulate genes associated with WNT signaling
		HEK293T			Reduce self-renewal
					Knockdown of NMDAR2B reduced breast-to-brain metastasis
				Glutamate Ionotropic Receptor NMDA Type Subunit 2B	
		MHCC97L			
	*In vivo/In vitro*	4 Oncopigs (A272, A273,	AXIN1	Axin 1	Knockout of AXIN1 or ARID1A on Proliferation and Migration
			ARID1A	AT-Rich Interaction Domain 1A	
		A274, and A343)			
		Porcine HCC cells			
[128]	*In vivo/In vitro*	Mice	SGOL1	Shugoshin 1	Decreased the cytotoxicity of sorafenib
		Huh7 SMMC-7721			
	*In vitro*	Huh7	KEAP1	Kelch Like ECH Associated Protein 1	KEAP1 inactivation deregulates KEAP1/Nrf2 pathway that contributes to drug resistance
[94]	*In vivo/In vitro*	HEK-293 Huh7	IQGAP1/FOXM1	IQ Motif Containing GTPase Activating Protein 1	Reverses sorafenib resistance
					Suppressing cancer stem cells
		LC9-293 HN3LC9-293			
				Forkhead Box M1	
		Huh7 xenografts			
	*In vivo/In vitro*	Mice	NCAPG	Non-SMC Condensin I Complex Subunit G	An essential oncogene
		HepG2 andSNU449			Tumor cell survival
		Huh7			
		HCCLM3			
[96]	*In vitro*	SNU449	AXL	AXL Receptor Tyrosine Kinase	Tyrosine kinase receptor
		HLF			
	*In vivo/In vitro*	Mice	EGFR	Epidermal Growth Factor Receptor	Regulating the EGFR-PI3K-AKT pathway
		HepG2			
		H22			Inhibit angiogenesis
[97]	*In vivo/In vitro*	Mice	MT genes	Metallothionein gene family	Chromosome conformation and chromatin immunoprecipitation
		Huh-7 HepG2			
	*In vivo/In vitro*	Mice	lncRNAs	Long Noncoding RNAs	Promoting G1/S progression
		MHCC97H			
		HEK293FT			

**Table 2:** A simplified list comparing CRISPR/Cas9 gene therapy with other common gene therapy approaches for HCC

Aspect	CRISPR/Cas9	Viral vectors	RNA interference
Efficiency	High, due to its ability to directly modify specific gene sequences, potentially correcting the genetic basis of HCC.	Variable; efficiency is often high but can depend on the type of virus used and its ability to infect liver cells.	High for silencing specific malignant genes. However, it doesn’t change the DNA sequence, so the treatment may need repetition.
Specificity	Very high; can be designed to target precise genetic locations, minimizing off-target effects.	Moderate; targets cell types based on the viral tropism. May infect non-target cells, causing off-target effects.	High; designed to silence specific mRNA molecules. Off-target effects can occur but are predictable and can be managed.
Safety	Relatively safe but not without concerns. Risks include off-target edits or immune responses.	Concerns include immune responses, toxicity, and potential for viral gene integration leading to mutagenesis.	Generally considered safe but may trigger immune responses or off-target effects.
Potential challenges	Delivery to cells, control of off-target effects, ethical/regulatory hurdles, and ensuring permanent and safe integration.	Efficient delivery, avoiding immunity, control over viral integration, and potential mutagenic effects.	Delivery mechanisms, transient effect requiring re-administration, and bypassing immune responses.

## Future Perspective

By editing cells *in vitro* and implanting them orthotopically, genetically tailored tumors can be generated for biomedical modeling. This approach holds great promise for the field of biomedical modeling. Porcine models can also be used to model other types of cancer. It is also possible to apply human gene-edited cells to other species using immunocompromised animals. The CRISPR delivery components can be optimized *in vivo* to develop pork gene editing models. Advances in the generation of animal models containing customized mutations will make it easier to develop and test precision medicine approaches.

Additionally, innovative CRISPR-based therapies can be examined in pigs via optimization and rigorous testing before being clinically tested in people. Even though CRISPR/Cas has the potential to be an effective therapeutic approach for genetic-based disorders, it remains unapplied to human patients for some reasons. The CRISPR/Cas9 technology will be improved by testing its therapeutic potential in animal models. The size, anatomical, and biological similarities of pigs make them an ideal animal model for testing the safety and effectiveness of CRISPR/Cas9.

Even though CRISPR/Cas9 has the potential to be an effective therapeutic approach for genetic-based disorders, it may remain unapplied to human patients for several reasons that it is still in the model evaluation phases.

It is becoming more nuanced and target-specific as CRISPR/Cas9-based gene therapy approaches HCC treatment extends beyond the preliminary findings within the current scope of research. Future research will likely focus on developing genetic alterations that will maximize the effectiveness of gene modification to inhibit HCC growth. For instance, knockouts of SNHG9 and CASC11 demonstrated these genes’ importance in proliferation and metastasis. A more pronounced therapeutic effect can be achieved by identifying more targeted genes and corresponding CRISPR strategies, moving beyond the monogenic approach to a possible polygenic process. The limitations of current HCC treatments, like sorafenib, make CRISPR/Cas9 technology a promising candidate to combine with existing or new drugs. Research needs to identify synergistic combinations to optimize gene editing’s therapeutic effects while minimizing off-target effects and resistance, a challenge observed in current regimens. Delivering genetic material to target cells effectively using nanoparticles has become increasingly difficult. These delivery vectors need to be improved in terms of specificity, stability, and safety. Biocompatible materials, targeted ligands, and controlled release mechanisms are likely to be studied more intensely, focusing on minimizing immunogenicity. Future endeavors must include designing rigorous clinical trials to test CRISPR/Cas9’s effectiveness, safety, and practicability for HCC since most research is still pre-clinical. There needs to be a systematic approach to capturing long-term outcomes, potential side effects, and improvements in quality of life for these trials. Patients with HCC exhibit significant genetic heterogeneity, making personalized gene therapy more effective. To achieve this, more research needs to be conducted on comprehensive genetic screenings and the development of CRISPR toolkits tailored to individual patient profiles that are quick and cost-effective.

Several limitations have been identified concerning CRISPR delivery methods for the treatment of HCC, which need to be addressed to improve clinical translation. Some of these limitations include 1) Target Specificity and Off-Target Effects, 2) Delivery Efficiency, 3) Immunogenicity, 4) *In Vivo* Editing Efficiency, 5) Gene Editing Control, and 6) Scale-Up and Manufacturing. The next generation of CRISPR/Cas9 systems for HCC therapy must combine advances in materials science, immunology, genomic analysis, and cellular biology to address these limitations. A successful clinical translation can be achieved with these improvements, which could provide precision, safety, and control.

The scientific community needs to come together to overcome these hurdles, from improving technical aspects to establishing rigorous ethical and regulatory frameworks. Towards the development of CRISPR into a reliable, widely applicable therapeutic approach, progress in these areas will be paramount.

Xenografts or organoids derived from patients are important preclinical models for HCC, which provide a better understanding of potential clinical outcomes before human trials. In addition to studying CRISPR system pharmacokinetics and pharmacodynamics, research should also involve a variety of animal models to reduce off-target effects. Minimizing off-target effects is essential for safety. Researchers need to use high-resolution genome-wide techniques to map off-target sites and understand unintended interactions within the hepatocyte genome. Moreover, safe delivery and transient expression strategies should also be prioritized to minimize unintentional genome editing by engineering advances to increase Cas enzyme fidelity. A comprehensive toxicological assessment will be crucial. Specifically, the liver is known for its vulnerability to gene therapy interventions, so CRISPR components should be evaluated for their immunogenicity, genotoxicity, and other systemic effects. The studies discussed identified specific lncRNAs and other genetic elements as influential in HCC. Through independent studies, we need to corroborate these findings, examining the therapeutic outcomes of manipulating these targets and establishing a clear connection between these targets and the pathogenesis of HCC. It is crucial to determine the most effective dose that minimizes risks. It involves optimizing delivery vectors for delivering hepatocytes while limiting non-target tissues. In addition, gene editing effects need to be explored for a longer period. To initiate human trials of CRISPR/Cas9 strategies for HCC, it is important to ensure they meet all regulatory requirements, including legal and ethical requirements for gene therapy.

The future of CRISPR/Cas9 gene editing in enhancing patient outcomes is cautiously optimistic when analyzing the evidence presented throughout research on HCC. However, it must navigate the precise channels of scientific, ethical, and clinical pathways. As a result, CRISPR/Cas9 is poised to lead to revolutionary advances in HCC treatment in the coming years. For gene editing to fully align with the real-world clinical environment and improve meaningful patient outcomes, rigorous research, ethical standards, and patient-centered approaches are necessary for this journey.

## Conclusions

An effective therapeutic strategy for HCC could be developed using the emerging gene-editing technology CRISPR/Cas9. The CRISPR-based approach aims to correct the genetic drivers of HCC pathogenesis, progression, and drug resistance by precisely targeting and editing genes involved in its pathogenesis, progression, and drug resistance. HCC cell lines and animal models have shown promising preclinical proof-of-concept results in preclinical studies. HCC models have demonstrated anti-tumor effects and inhibition of cancer growth when specific oncogenes, tumor suppressors, and genes related to metastasis, proliferation, and chemoresistance have been knocked out or edited. It is possible to tailor gene modifications to target molecular mechanisms underlying HCC through CRISPR, as demonstrated by targeting CTNNB1, PDHA, and DUSP4. Many limitations and challenges will need to be addressed before clinical translation. We need to optimize delivery methods to maximize tumor cell targeting, minimize off-target effects, and ensure safety. A personalized approach may also be necessary for HCC because of the genetic heterogeneity of the disease. CRISPR may provide better outcomes than a standalone strategy when combined with chemotherapy, immunotherapy, or nanoparticles. CRISPR/Cas9 gene editing is still in its early experimental stages but is set to become a future pillar of HCC treatment due to its versatility and precision. Conducting rigorous preclinical research and phased clinical trials is crucial to determining real-world viability and ethical issues. There is tremendous potential for CRISPR/Cas9 to advance HCC therapies, but making cutting-edge genetic tools available to patients requires further multidisciplinary research. As a result, gene editing with CRISPR/Cas9 has proven therapeutic for the complex genetic drivers of HCC, but converting these proofs-of-concept into clinical treatments requires systematic innovation and evaluation in the long run. CRISPR will succeed in the lab but cannot be used in the clinical setting without collaboration across scientific, regulatory, and ethical domains.

## Data Availability

Not applicable.
